# Challenges and recommendations for implementation of oncology clinical practice guidelines in China

**DOI:** 10.1371/journal.pmed.1005261

**Published:** 2026-10-28

**Authors:** Minzhi Lv, Duanduan Yuan, Yue Suo, Fang Ding, Lijuan Hu, Lei Cao, Yi Wang, Jing Lei, Shenglan Tang, Tianshu Liu

**Affiliations:** 1 Department of Cancer Screening and Prevention, Zhongshan Hospital, Fudan University, Shanghai, China; 2 Cancer Centre, Zhongshan Hospital, Fudan University, Shanghai, China; 3 Center of Evidence-based Medicine, Fudan University, Shanghai, China; 4 Innovation Lab for Cancer Prevention and Control (CALab), Duke Kunshan University, Kunshan, Jiangsu, China; 5 School of Public Health, Fudan University, Shanghai, China; 6 National Health Commission Key Laboratory of Health Technology Assessment, Fudan University, Shanghai, China; 7 Department of Medical Affairs, Zhongshan Hospital, Fudan University, Shanghai, China; 8 Duke Global Health Institute, Duke University, Durham, North Carolina, United States of America; 9 Global Health Research Center, Duke Kunshan University, Kunshan, Jiangsu, China; 10 Department of Population Health Sciences, Duke University School of Medicine, Durham, North Carolina, United States of America; 11 Duke–NUS Medical School, Singapore, Singapore

## Abstract

- As China’s cancer burden continues to rise and treatment becomes increasingly complex, clinical practice guidelines (CPGs) are crucial to standardising cancer care and supporting clinical decision-making. Over the past decades, China has made substantial progress in developing oncology CPGs, transitioning from expert consensus and international adaptation toward domestically led guideline production.
- This Policy Forum presents an analysis of the optimisation and implementation of oncology CPGs in China. We argue that the core challenge facing oncology CPGs in China is not a lack of guidelines, but the absence of an integrated governance and implementation ecosystem, and insufficient alignment with local evidence.
- By situating China's oncology sector within a macro-health-system perspective, this Policy Forum provides a conceptual blueprint for other transition economies experiencing a mismatch between rapid clinical technology scaling and fragmented regulatory governance.
- We propose a four-part strategic framework: (1) Establishing a national joint clearinghouse and registration system; (2) integrating resource-stratified pathways with locally relevant evidence; (3) expanding free, diversified dissemination channels; and (4) embedding implementation science alongside healthcare financing alignment.
- Addressing these systemic gaps offers scalable lessons for global health systems aiming to transition from the quantity-driven expansion of clinical guidelines to equity-oriented, system-integrated quality enhancement.

## Abstract 2

In this This Policy Forum, Minzhi Lv, Duanduan Yuan, Yue Suo and colleagues argue that the core challenge for implementation of oncology clinical practice guidelines in China is not a lack of guidelines, but the absence of an integrated governance and implementation ecosystem, and propose a four-part strategic framework for addressing these issues.

## Introduction

Cancer remains a major global public health challenge in China and globally. In 2022, approximately 20 million new cancer cases and nearly 9.7 million cancer deaths occurred globally, with China accounting for approximately one quarter of this burden [1]. At the same time, the rapid expansion of targeted therapy, immunotherapy, and other emerging treatment modalities has substantially increased the complexity of decision-making in oncology [2]. Thus, clinical practice guidelines (CPGs) have an increasingly important role to play in synthesising evidence, reducing unwarranted variation, and supporting equitable care [3].

Oncology CPG development in China started relatively late, initially relying on expert consensus and adaptation of international guidelines before shifting toward domestically led production. Over the past decade, China has experienced rapid growth in the number and scope of oncology CPGs, with notable progress in both quantity and quality (see Fig 1). Guideline coverage has expanded across tumour types and increasingly incorporated precision medicine and whole‑course management [4,5]. Furthermore, this growth has been accompanied by a rapid surge in high-quality domestic evidence. However, rapid growth has not resolved persistent variation in cancer care. Guideline development remains fragmented across issuing organisations, methodological quality and updating are uneven, locally relevant evidence is not consistently incorporated, and dissemination and implementation remain limited across clinical settings.

**Fig 1:**
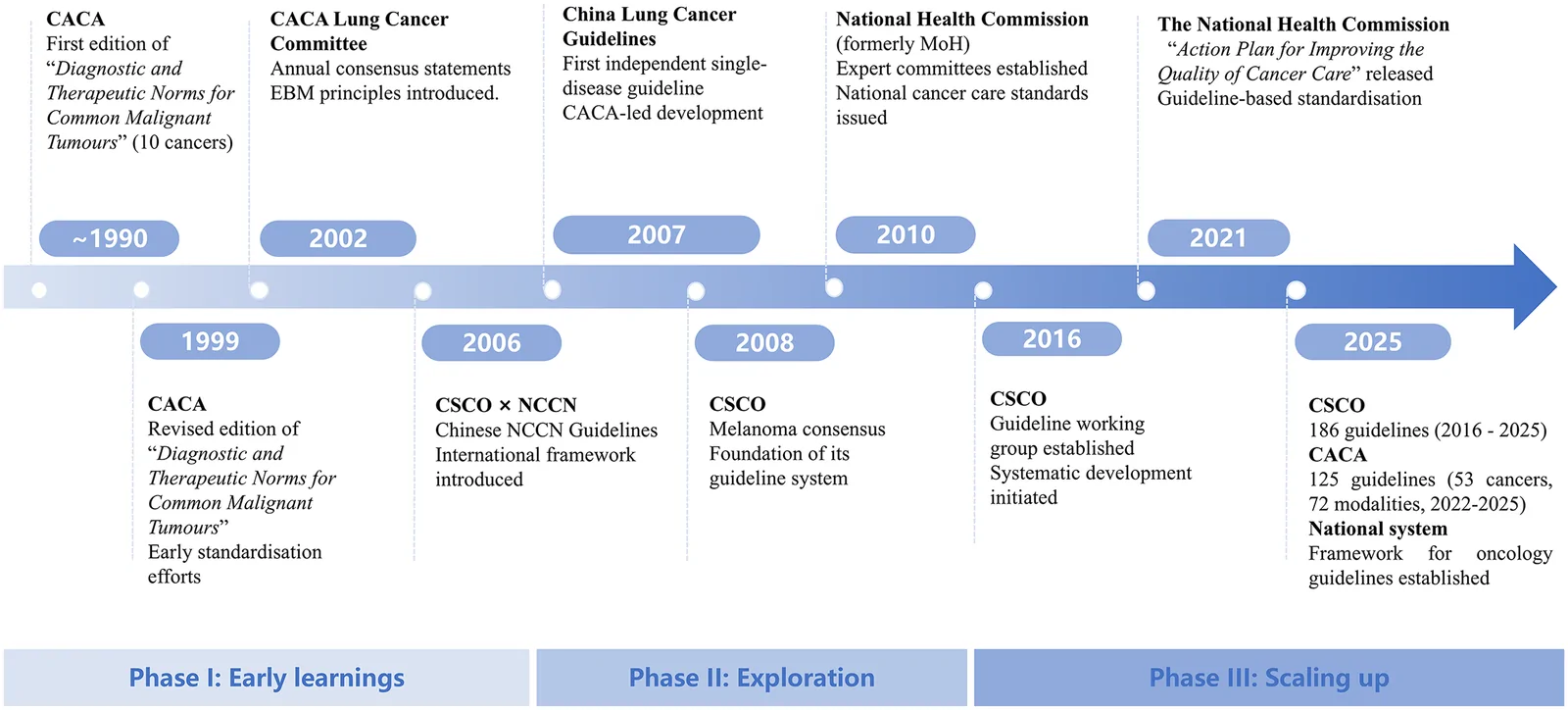
Timeline of the development of oncology clinical practice guidelines in China. CACA, China Anti-Cancer Association; CSCO, Chinese Society of Clinical Oncology; EBM, evidence-based medicine; MoH, Ministry of Health; NCCN, National Comprehensive Cancer Network.

We argue that the central challenge is not a shortage of oncology guidelines, but the lack of an integrated governance and implementation system linking guideline development, dissemination, and clinical use. This analysis examines these system-level barriers and proposes policy priorities to strengthen guideline quality, accessibility, and impact in China. China’s experience may also offer lessons for other middle-income health systems facing rapid technological change, fragmented service delivery, and persistent inequities in cancer care.

### Fragmentation at the core: Challenges in the development of oncology CPGs in China

Over the past decade, the number of oncology CPGs developed in China has increased substantially [6,7], reflecting growing attention to evidence-based medicine and clinical standardisation. However, this quantitative expansion has been accompanied by increasing disintegration in guideline development. We identified that the absence of a national-level coordinating and governance mechanism represents the most fundamental constraint undermining the coherence, credibility, and effectiveness of oncology CPGs in China.

In the current system, guidelines are developed by the National Health Commission (NHC) and major professional societies, such as the Chinese Society of Clinical Oncology (CSCO), the China Anti-Cancer Association (CACA), and branches of the Chinese Medical Association (CMA), each of which issues its own guidelines. A systematic review of 2,654 Chinese CPGs found that 71.1% were developed by medical specialty societies and only 2.6% by government agencies [6]; in oncology, CSCO published 186 guidelines between 2016 and 2025, while the CACA guideline programme (2022–2025) covered 53 cancer types and 72 treatment modalities, totalling 125 guidelines. Although this pluralistic model has encouraged disciplinary engagement, the parallel development of oncology guidelines reflects historically evolved, partly overlapping roles among the NHC, CSCO, CACA, and branches of the CMA. NHC-issued guidance is more directly linked to administrative implementation and medical quality control, whereas society-led guidelines derive authority primarily from professional consensus and clinical uptake. However, their functional boundaries and hierarchy remain insufficiently defined, and overlapping mandates may reinforce incentives to maintain parallel guideline systems. Consequently, guidelines addressing the same type of cancer may coexist with differences in scope, treatment pathways, and evidence interpretation, creating uncertainty for clinicians and reinforcing fragmentation in the lack of formal coordination.

This lack of integration also contributes to variability in methodological rigour and transparency. While some oncology guidelines in China demonstrate methodological quality comparable to international standards, evaluations using AGREE II and the RIGHT reporting checklists consistently show wide variation across tumour types and issuing organisations [8–20]. Some guidelines rely on insufficient or low‑quality evidence, leading to inappropriate recommendations—a challenge not unique to China. The analyses of American Society of Clinical Oncology (ASCO) guidelines have also identified strong recommendations supported by low-quality evidence, particularly in consensus-based recommendations [21]. In China, many guidelines lack systematic evidence grading using the Grading of Recommendations Assessment, Development and Evaluation (GRADE) framework [22] or transparent evidence synthesis. Non‑evidence‑based consensus statements, often developed using less rigorous methodology, further compound concerns about the quality of the evidence base [6].

Oncology guideline developers have also used heterogeneous systems for grading evidence and formulating recommendation strength, which may complicate cross-guideline interpretation and clinical application. Harmonisation of core methodological standards could facilitate greater consistency and comparability across guidelines, while allowing individual organisations to retain context-specific approaches that are aligned with resource availability and clinical applicability.

In addition, gaps in high-quality domestic evidence remain particularly important for digestive cancers that impose a substantial disease burden in China, including gastric and hepatobiliary malignancies. In biliary tract cancers, for example, most of the China-specific evidence on systemic and emerging combination therapies comes from relatively small retrospective or real-world cohorts, while prospective data on some targeted and immune therapies remains limited [23–26]. These gaps constrain the development and timely updating of robust, locally relevant guidelines.

Structural misalignment between guideline development and real-world clinical contexts further undermines applicability. Recommendations are sometimes overly broad or vague, lack supportive tools for clinical implementation, or are insufficiently informed by local data [27,28]. Many guidelines are based primarily on the practice of large urban hospitals, with limited consideration of resource‑constrained settings, thus reducing applicability at lower levels of care [28,29]. In China, improving guideline applicability at the county/district level should not be equated with decentralising all oncology interventions. Although national policies increasingly emphasise strengthening and standardising cancer care at the county/district level, the feasibility of implementing specific recommendations may depend on their institutional capabilities. This is particularly important in surgical oncology, where complex cancer surgery depends not only on surgeon expertise but also on anaesthesia, operating-room infrastructure, pathology, perioperative care, and multidisciplinary support. Limited access to advanced staging, radiotherapy, molecular testing, and Multi-Disciplinary Team (MDT) services may further restrict guideline-recommended multimodal pathways surrounding surgery. Resource-adapted guidelines should therefore distinguish interventions that can be safely standardised and delivered at county-level hospitals from complex procedures requiring defined capability thresholds and referral to higher-level centres. Although some professional groups have begun developing resource-adapted pathways for county-level practice, these initiatives remain fragmented and are not systematically integrated into national guideline frameworks [30–33].

Timely updating represents another persistent weakness in guideline development. Internationally, guideline revision cycles range from one to five years, although many organisations do not specify clear schedules [34,35]. Historically, China’s guideline update rate has been low: as of December 2012, only 10% of 380 guidelines had ever been revised, with average update intervals exceeding five years [36,37]. This situation has improved in recent years, particularly in oncology, where major guidelines—such as those issued annually by CSCO—are updated more frequently [38–40]. Nevertheless, some guidelines remain outdated. Limited institutional continuity, absence of sustained oversight mechanisms, and lack of standardised updating methodologies continue to impede timely revision [41].

Finally, transparency and standardisation of conflict‑of‑interest management remain insufficient. AGREE II assessments show low scores in the domain of editorial independence and conflict‑of‑interest disclosure [10,13,18]. A systematic review of 2,654 Chinese CPGs found that only 18.6% reported the conflicts of interest, only 1.36% described methods for collecting and reviewing such information, and none provided publicly accessible conflict-of-interest statements for all authors [9]. These findings suggest that gaps extend beyond disclosure itself to the standardisation of conflict identification, review and management procedures, public accessibility of individual declarations, and independent oversight. More consistent disclosure requirements, predefined management procedures, and independent review are therefore needed to strengthen the credibility and editorial independence of guideline recommendations.

Taken together, these challenges are not isolated technical deficiencies but interrelated consequences of a fragmented development model. Without authoritative coordination, unified methodological standards, and sustained oversight, improvements in guideline quantity alone are unlikely to translate into consistent quality or clinical impact. Addressing fragmentation at the development stage is therefore a prerequisite for strengthening dissemination, implementation, and ultimately the effectiveness of oncology CPGs in China.

### From guidelines to practice: The persistent knowledge-to-practice gap

The impact ofoncology CPGs depends not only on their quality but also on their effective dissemination and implementation. In China, however, a substantial gap persists between guideline publication and routine clinical practice. This knowledge-to-practice gap represents a critical bottleneck, limiting the real-world impact of oncology CPGs and contributing to persistent variation in cancer care and treatment.

#### Dissemination barriers.

In China, guideline dissemination relies mainly on official websites, academic conferences, journal publications, and printed books. While these formats are effective for specialists in large urban hospitals, they offer limited reach and high access barriers for clinicians in primary and secondary care settings. Conferences are constrained by scale and geography, journals are often behind paywalls, and printed materials are costly and quickly outdated. Even when guidelines are available online, fragmented and poorly indexed platforms make them difficult to locate and use. From a policy perspective, this reflects a dissemination model that remains provider-centred and publication-oriented, rather than aligned with the needs and workflows of frontline clinicians.

These limitations disproportionately affect primary-level healthcare institutions. Clinicians in these settings show low awareness and utilisation of guidelines [27,42], relying largely on informal access routes such as institutional distribution or public search engines. In oncology, this gap is particularly evident: despite the availability of multiple domestic guidelines, awareness of international National Comprehensive Cancer Network guidelines exceeds 90% in a colorectal cancer-specific survey [20]. This imbalance reflects not only differences in perceived authority but also disparities in dissemination strategies, format accessibility, and supporting educational resources.

Compared with major international organisations, Chinese guideline developers have been slower to adopt diversified dissemination approaches such as mobile platforms, interactive tools, and patient-oriented materials (Table 1). As a result, the guideline content remains largely static and insufficiently integrated into clinical workflows. Taken together, these findings suggest that dissemination in China not only limited in reach, but structurally disconnected from implementation, constraining the real-world impact of oncology guidelines.

**Table 1:** Comparative analysis of guideline ecosystems, dissemination models, and regulatory enforcement across major oncology organisations.

Guideline and Setting	Access policy and digital infrastructure	Educational and professional outreach	Patient-centered adaptation	Regulatory alignment and policy enforcement level
**NCCN(USA)**	**Free registration;** highly interactive online portals, dynamic digital search tools, and dedicated mobile apps.	Global certification programs, interactive webinars, and standardised Continuing Medical Education (CME) courses.	**Extensive;** translated into multiple languages and customised for specific patient education literacy.	**Market-driven mandate;** systematically bound to private commercial insurance coverage policies and Medicare/Medicaid reimbursement pathways.
**ASCO(USA)**	**Paywalled/member-restricted;** integrated with advanced digital assistants (e.g., Google AI guideline assistant) and mobile apps.	Direct integration into annual international meetings, certified oncology CME credits.	**High;** dedicated patient guides and educational toolkits developed via the *Cancer.Net* platform.	**Quality-linked incentive;** serves as the benchmark for federal quality-based physician payment programs (e.g., MIPS, QOPI).
**ESMO(Europe)**	**Open Access** pocket guidelines, dynamic web tools, slide kits, and standalone mobile platforms.	Structured European CME credits, specialised oncology fellowship workshops.	**High;** comprehensive, consumer-friendly patient versions available across major malignancy types.	**Regional adaptation;** formally endorsed or adapted by individual European national health authorities to guide regional public funding.
**NICE(UK)**	**Full public Open-Access;** fully integrated interactive pathways embedded into the National Health Service (NHS) digital architecture.	Shared learning platforms, localised implementation toolkits, and clinical audit tools.	**Mandatory;** strictly co-produced with patient advocacy groups; highly transparent public versions.	**Statutory mandate;** legally linked to NHS clinical commissioning groups, public funding mandates, and local quality premium payouts.
**CSCO(China)**	**Restricted access (membership and print-purchase);** printed annual books, free PDF downloads for active members, pilot AI decision-support interfaces (CSCO AI).	Large-scale annual national conferences and widespread traveling educational workshops.	**Pilot phase;** localised adaptations for limited major tumour types (e.g., CSCO NSCLC patient version).	**Advisory;** broadly adopted clinical voluntary benchmark, with indirect evidentiary reference value for national reimbursement drug list pricing and DRG/DIP formulation, yet lacking legally binding linkage to national reimbursement or hospital payment adjustment rules.
**CACA(China)**	**Print-purchase model;** large-scale printed books and compilations, supplemented by static official web hosting.	Massive-scale nationwide travelling educational workshops and specialised sub-society seminars.	**Largely underdeveloped;** only general public science materials are provided without guideline-matched patient versions.	**Advisory;** independent academic society consensus document with no statutory mandatory implementation rules, nor formal integration into national public hospital performance appraisal metrics, though officially endorsed for national cancer standardisation initiatives.
**National Health Commission(China)**	**Free public access (official web download);** published in static text or PDF formats, though clinical coverage across specific disease types remains relatively narrow.	Disseminated and monitored primarily through hierarchical national and provincial medical quality control centers.	**Absent;** exclusively designed as administrative and clinical reference documents.	**Semi-statutory/baseline;** serves as the authoritative baseline framework for administrative medical dispute resolutions and hospital tier-3 inspections, but lacks real-time alignment with healthcare financing metrics.

CACA, China Anti-Cancer Association; CSCO, Chinese Society of Clinical Oncology; NCCN, National Comprehensive Cancer Network; ESMO, European Society for Medical Oncology; NICE, National Institute for Health and Care Excellence; QOPI, Quality Oncology Practice Initiative; MIPS, Merit-Based Incentive Payment System.

#### Implementation barriers.

Even when high‑quality guidelines are well developed and disseminated, effective translation of evidence into practice is not guaranteed [43]. In China, guideline implementation remains constrained by persistently low adherence, contributing to overtreatment, inconsistent care, and variability in outcome [44,45].

Available evidence from China and other settings suggests substantial gaps between recommendations and practice [46–56]. For example, a multi-center study of 1,828 patients with non-small cell lung cancer across 20 hospitals in Liaoning Province found that only 36.7% received both guideline-concordant diagnosis and treatment, which was associated with a 29% lower risk of lung cancer mortality [56]. These patterns indicate that the challenge is not simply awareness of guidelines, but the absence of mechanisms put in place to support and enforce their use in practice.

Barriers to implementation operate at multiple levels. For clinicians, heavy workloads, limited formal training in evidence-based medicine, and entrenched practice habits constrain the ability to interpret and apply guideline recommendations [27,29,57,58]. Although most clinicians’ attitudes toward cancer guidelines are generally positive [58,59], concerns persist that guidelines may promote “cookbook medicine” or oversimplify complex decisions [60–62]. Patient-level factors, including limited understanding of treatment benefits and risks, financial constraints, and treatment refusal, further complicate adherence [53]. However, these individual-level barriers are reinforced by systemic weaknesses.

At the systemic level, guideline implementation is hindered by a regulatory oversight void and misaligned policy incentives. Most oncology CPGs remain purely advisory, as existing health quality-assurance bodies focus on basic safety rather than systematic evaluation of guideline adherence. This accountability gap is further complicated by structural tensions with healthcare financing mechanisms, notably the National Tertiary Public Hospital Performance Appraisal [63] and Diagnosis-Related Group (DRG)/Diagnosis-Intervention Packet (DIP) payment models [64]. DRG and DIP are case-based prospective payment approaches that reimburse hospitals according to predefined patient groupings and payment standards, thereby creating strong incentives to contain costs and improve resource efficiency. Because these cost-containment mechanisms are not necessarily aligned with clinical guideline recommendations, strict adherence to advanced, resource-intensive guideline recommendations may place hospitals under financial or performance pressure. Consequently, the lack of a unified framework integrating quality monitoring with cost control forces clinicians into a pragmatic compromise between clinical excellence and administrative compliance.

Together, these factors contribute to a persistent knowledge-to-practice gap, in which oncology guidelines are increasingly available yet insufficiently utilised, particularly outside tertiary centres. This paradox underscores that expanding guideline production alone is unlikely to improve outcomes without embedding dissemination and implementation within a coordinated system. Addressing this gap therefore requires moving beyond publication-centred approaches toward an integrated model that treats dissemination and implementation as core components of the guideline ecosystem, rather than downstream afterthoughts.

### Policy recommendations for implementing oncology CPGs in China

This analysis draws on a synthesis of published literature, guideline documents, and policy reports to identify key challenges and inform strategic recommendations. Based on the above analysis, we propose four strategies aimed to improve the effectiveness of oncologic care in China (Fig 2). The recommendations outlined below are formulated not merely to remediate China's localised bottlenecks, but to propose a replicable framework for health system strengthening in similar developmental contexts.

**Fig 2:**
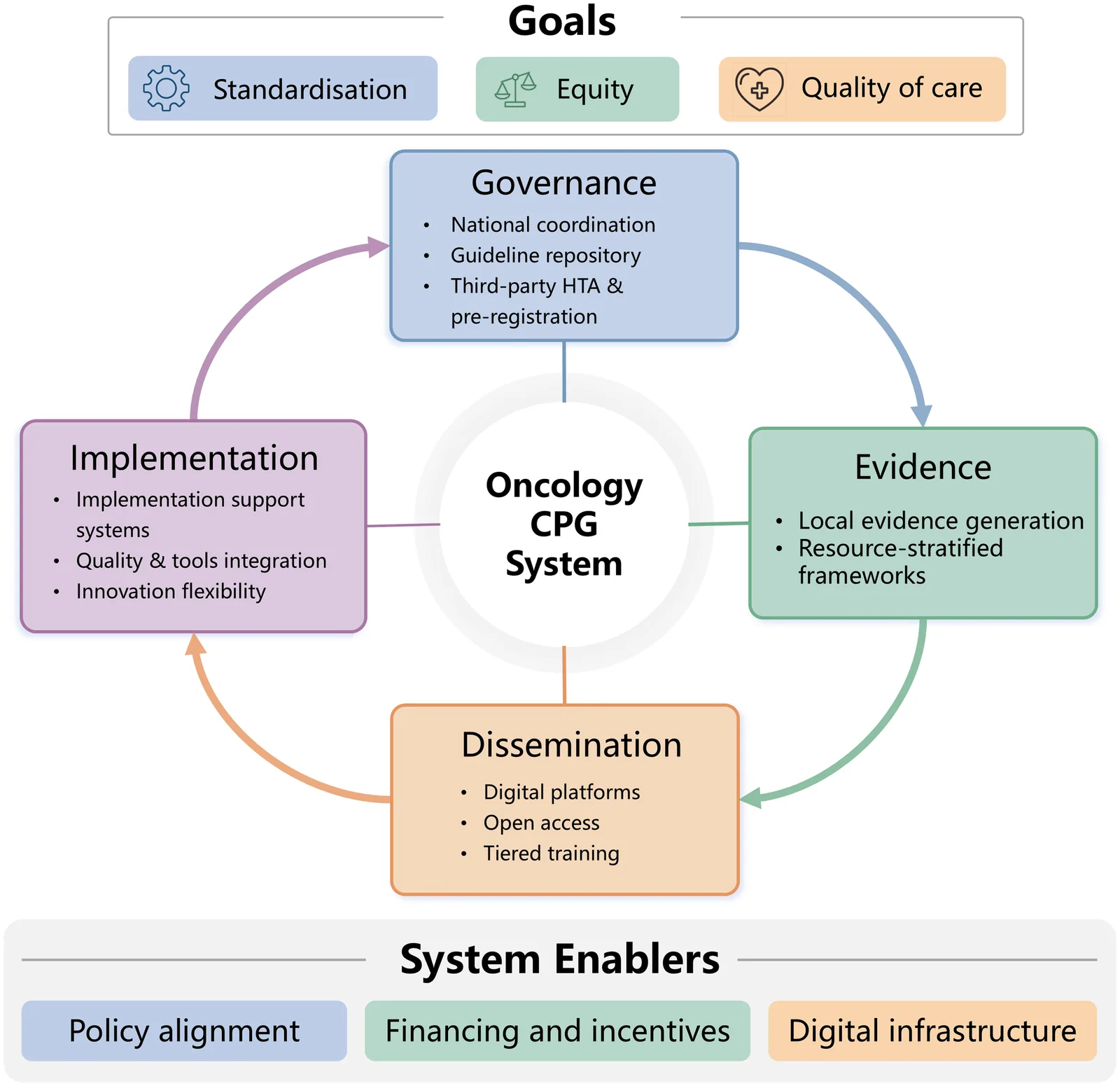
A system-integrated governance framework to bridge the guideline-implementation paradox. This framework conceptualises the structural alignment required to transform clinical practice guidelines from static scientific documents into dynamic quality-improvement tools. It illustrates the multi-level interplay between unified national oversight (e.g., centralised registration and third-party evaluation), resource-stratified evidence synthesis tailored to regional disparities, and systemic integration with healthcare financing mechanisms (such as adjusting DRG/DIP payment capping). Crucially, the framework advocates for flexible clinical governance that balances standardised care delivery in primary settings with evidence-based breakthrough innovation at elite medical centres. CPG, clinical practice guideline; HTA, Health Technology Assessment.

#### Strategy 1: Establish a national joint clearinghouse and registration system for oncology guidelines (Short- to medium-term priority).

Led by the NHC, a national coordinating mechanism should be put place to oversee oncology guideline development and facilitate coordination among the CMA, CSCO, CACA, and other major relevant organisations. A unified national platform should require prospective registration of guideline topics and planned updates, enabling early identification of overlapping initiatives and coordination of scope and responsibilities before development begins. Parallel development on the same topic should require explicit justification based on distinct target populations, clinical settings, or intended functions, thereby reducing unnecessary duplication while allowing justified parallel development. The coordinating mechanism should also promote harmonised methodological standards for evidence grading and recommendation formulation across issuing organisations.

Rather than relying on unstructured repositories, the National Center for Medical Quality Control should oversee an open-access National Oncology Guideline Clearinghouse—a centralized platform for systematically collecting, organizing, and disseminating up-to-date oncology guidelines. This platform should employ “living guideline” approaches to support timely and dynamic updating [65]. Independent third-party methodological evaluation bodies should conduct regular assessments of guideline quality, and publish findings in order to strengthen methodological transparency and conflict-of-interest accountability. The platform should also establish explicit sunset and decommissioning criteria, ensuring that outdated or superseded guidelines are formally withdrawn from active use and clearly linked to their current replacements.

#### Strategy 2: Integrate resource-stratified pathways and local high-quality evidence into guideline synthesis (Medium- to long-term priority).

To bridge the urban-rural care gap, future oncology CPGs must move beyond monolithic recommendations toward a resource-stratified framework (categorised into basic, core, and enhanced pathways). Such stratification should define not only feasible local alternatives, but also minimum institutional capabilities and explicit referral thresholds for complex interventions. Guided by clinicians from county-level and resource-constrained settings, together with patient representatives [66], guidelines should explicitly define operational alternatives based on regional diagnostic and treatment capacities and local reimbursement realities. For example, in resource-constrained settings lacking advanced imaging infrastructure, standardised CT-based diagnostic and staging protocols must be structurally legitimised as acceptable alternatives rather than sub-optimal choices. Building on a growing surge of domestic high-quality evidence, dedicated public funding should prioritise local prospective studies, empowering indigenous data to continuously refine and localise future oncology guidelines.

#### Strategy 3: Expand free and diversified dissemination channels and strengthen tiered training to enhance guideline uptake (Short-term priority).

To improve the reach and use of CPGs, dissemination strategies should prioritise free access and diversified delivery channels. Using the clearinghouse proposed in Strategy 1 as the dissemination backbone would ensure barrier-free access for all healthcare professionals, particularly those in primary and resource-limited settings. At the same time, greater use of digital platforms—such as professional mobile applications, online education platforms, and widely used social media channels—can facilitate timely and convenient access to guideline content across clinical settings. Emerging digital tools, including artificial intelligence-based clinical decision-support systems, may further support real-time guideline consultation during routine care. CSCO has already begun exploring such systems [67,68].

In parallel, tiered and role-specific training programs should be strengthened to enhance clinicians’ capacity to implement guideline-recommended care. Training for primary-level providers should focus on core clinical pathways, key recommendations, and practical application, thereby lowering learning barriers and promoting more consistent integration of evidence-based practices into everyday clinical decision-making.

#### Strategy 4: Embed implementation science into routine oncology care and align quality metrics with healthcare financing (Medium- to long-term priority).

To bridge the persistent knowledge-to-practice gap, health authorities, hospital governance bodies, and professional societies must transition from passive guideline dissemination to active implementation science frameworks. This shift requires integrating structured implementation planning directly into the guideline lifecycle—encompassing systematic pre-development barrier assessments, targeted behavioural intervention designs, and continuous outcome monitoring—by adopting validated toolkits such as CAN-IMPLEMENT, a framework for guideline adaptation and implementation planning [69]. Systematic post-implementation evaluation should further assess whether guideline adoption improves patient survival and other clinical outcomes, as well as its effects on healthcare utilisation and costs, with these findings feeding back into subsequent guideline updates or decommissioning decisions.

Crucially, clinical quality metrics must be harmonised with macro-level health system reforms without imposing rigid conformity. To mitigate friction between clinical excellence and fiscal control, the NHC and the National Healthcare Security Administration should co-develop adaptive payment mechanisms within DRG/DIP frameworks, including specialised reimbursement pathways for complex cases requiring resource-intensive care or justified deviations from standard CPGs. Concurrently, the National Tertiary Public Hospital Performance Appraisal should pivot from raw cost-containment toward quality-adjusted, value-based metrics. Rather than blindly penalising clinical variance, these assessments must accommodate evidence-based clinical innovation, particularly at elite medical centres tackling highly complex clinical scenarios. Structurally reconciling financial regulation with flexible clinical governance transforms guidelines from administrative dogmas into incentivised, dynamic benchmarks that successfully balance care standardisation with breakthrough innovation.

## Conclusions

China’s oncology guideline system has advanced rapidly, but future progress will depend less on producing more guidelines than on strengthening governance across the guideline lifecycle. Greater coordination among issuing organisations, stronger integration of locally relevant evidence, and resource-sensitive implementation are needed to improve methodological consistency, clinical feasibility, and real-world impact. These reforms should balance national oversight with professional autonomy while aligning guideline implementation with existing quality, financing, and referral systems. Further empirical research is needed to evaluate cancer-specific and regional differences in guideline implementation and outcomes.

## Abbreviations

ASCO: American Society of Clinical Oncology
CACA: China Anti-Cancer Association
CMA: Chinese Medical Association
CME: Continuing Medical Education
CPGs: clinical practice guidelines
CSCO: Chinese Society of Clinical Oncology
DIP: Diagnosis-Intervention Packet
DRG: Diagnosis-Related Group
EBM: evidence-based medicine
ESMO: European Society for Medical Oncology
GRADE: Grading of Recommendations, Assessment, Development and Evaluation
HTA: Health Technology Assessment
MDT: Multi-Disciplinary Team
MIPS: Merit-Based Incentive Payment System
MoH: Ministry of Health
NCCN: National Comprehensive Cancer Network
NHC: National Health Commission
NHS: National Health Service
NICE: National Institute for Health and Care Excellence
QOPI: Quality Oncology Practice Initiative
